# Genomic potential of crustose coralline algae-associated bacteria for the biosynthesis of novel antimicrobials

**DOI:** 10.1099/mgen.0.001456

**Published:** 2025-07-25

**Authors:** Diego Lera-Lozano, Jordan S. Ruiz-Toquica, Samantha A. Kratman, Matthew W. Holt, Clancy A. McIntyre, Elizabeth K. Jones, Mateo Lopez-Victoria, Kim B. Ritchie, Mónica Medina, Raúl A. González-Pech

**Affiliations:** 1 Department of Biology, The Pennsylvania State University, University Park, PA, USA; 2 Department of Ecology and Evolutionary Biology, University of California Los Angeles, Los Angeles, CA, USA; 3 Faculty of Natural Sciences and Engineering, Universidad de Bogotá Jorge Tadeo Lozano, Bogota, Colombia; 4 Department of Biology and Marine Biology, University of North Carolina Wilmington, Wilmington, NC, USA; 5 Schreyer Honors College, The Pennsylvania State University, University Park, PA, USA; 6 Department of Natural Sciences, University of South Carolina Beaufort, Beaufort, SC, USA; 7 Department of Natural Sciences and Mathematics, Pontificia Universidad Javeriana, Cali, Colombia; 8 Department of Biology, Texas State University, San Marcos, TX, USA

**Keywords:** antimicrobial activity, bioprospecting, crustose coralline algae, crustose coralline algae (CCA) bacteria, microbial ecology

## Abstract

The global rise of antimicrobial resistance has intensified efforts in bioprospecting, with researchers increasingly exploring unique marine environments for novel antimicrobials. In line with this trend, our study focused on bacteria isolated from the unique microbiome of crustose coralline algae (CCA), which has yet to be investigated for antimicrobial discovery. In the present work, bacteria were isolated from a CCA collected from Varadero Reef located in Cartagena Bay, Colombia. After performing antimicrobial assays against antibiotic-resistant human and marine pathogens, three isolates were selected for genome sequencing using the Oxford Nanopore technology. Genome mining of the high-quality assemblies revealed 115 putative biosynthetic gene clusters (BGCs) and identified genes in relevant biosynthetic pathways across the three genomes. Nonetheless, we hypothesize that the biosynthesis of antimicrobial compounds results from the expression of undescribed BGCs. Further analysis revealed the absence of genes pertaining to the synthesis of coral larvae settling molecule tetrabromopyrrole, commonly produced by CCA-associated bacteria. We also discuss how differential representation of gene functions between the three isolates may be attributed to the distinct ecological niches they occupy within the CCA. This study provides valuable resources for future research aimed at the discovery of novel antimicrobials, particularly in the face of the antibiotic-resistance global crisis, and highlights the potential of specialized marine environments like CCA.

**Impact Statement**

Previous research has identified the ocean and its microorganisms as rich sources of novel natural products, offering promising solutions to the escalating crisis of antimicrobial resistance. Among these, bacteria associated with reef organisms, particularly crustose coralline algae, have garnered significant interest due to the production of unique metabolites, such as those that facilitate larval settlement in marine organisms. However, their potential antimicrobial properties remain unexplored. Our study investigates the antimicrobial activity of bacterial isolates from a crustose coralline alga, laying the groundwork for future bioprospecting efforts aimed at discovering novel antibiotics.

## Data Summary

Raw sequencing data, genome assembly and annotation are available under the BioProject accession number PRJNA1207476.

## Introduction

Antimicrobial resistance poses a critical threat to public health globally, with estimates suggesting that by 2050, it could claim up to 10 million lives annually and cause substantial economic losses, impacting healthcare and human development [1]. In response to this growing crisis, the urgent need for new antimicrobials, particularly novel structural classes that can circumvent existing resistance mechanisms, has become paramount [2]. Considering this, bioprospecting efforts have intensified in pursuit of new antimicrobial compounds with the potential to target antimicrobial-resistant pathogens.

Over the past century, scientists have identified natural products with a wide variety of use cases, including agricultural implementations (e.g. biopesticides), nutraceuticals (e.g. dietary supplements) and, notably, pharmaceuticals (e.g. antibiotics). Bioprospecting has been critical in these discoveries. In particular, bioactive secondary metabolites have been identified from bacteria living in environments with a high microbial diversity due to their broad metabolic spectra [3]. For example, the commonly used antibiotic vancomycin was originally recovered from a soil bacterium [4]. Similarly, the antiviral scytovirin, used to treat HIV, was derived from the freshwater cyanobacterium *Scytonema varium* [5]. Bacteria living in marine sediments contributed to the establishment of the antitumour antibiotics marinomycins [6].

Coral reefs are composed of several environmental niches that support a diverse array of both macro- and micro-organisms. Recent research has revealed that these ecosystems harbour a rich microbial community compared to many other environments [7]. This microbial richness is characterized by substantial heterogeneity in community composition and metabolic potential across different microenvironments within a single coral reef [8]. Microbial communities associated with reefs have been identified as promising sources of antimicrobial compounds. For example, an initial screening of culturable bacterial strains from the elkhorn coral (*Acropora palmata*) revealed that 20% exhibited antibiotic activity [9]. Additionally, coral-associated bacteria, like *Pseudovibrio* sp. P12, synthesize compounds such as tropodithietic acid, which effectively inhibits various coral pathogens resistant to commercial antibiotics [10].

While reef organisms like sponges and soft corals have provided notable antibiotics including halichondrin B [11] and eleutherobin [12], respectively, microorganisms associated with other reef organisms have been relatively underexplored for novel antimicrobials. A prime example is crustose coralline algae (CCA). CCAs host communities of bacteria with diverse functional roles. For example, abundant members of CCA microbial communities can include specialized, host-adapted bacteria [13] or common photoautotrophs [14], so there appears to be a range of metabolic interdependence between the microbes and with the algal host [15]. Stressors, such as heat, can cause shifts in the CCA microbiome [16] that likely relate to differential tolerance ranges among microbial taxa. These diverse bacterial communities thus bear great biotechnological potential [17,19]. These bacteria produce unique metabolites that contribute to host defence and health. While some CCA-associated bacteria like *Propionibacterium* and *Planomicrobium* show antimicrobial activity [20,22], research has primarily focused on their role in coral larval settlement [23,24] and antifouling properties [25]. The rich microbial diversity and metabolite production in CCA microenvironments offer promising opportunities for novel antimicrobial discovery.

Biosynthetic gene clusters (BGCs) play a crucial role in microbial adaptation and survival. They regulate the production of secondary metabolites, i.e. compounds not directly involved in the growth or reproduction of microorganisms but essential for their environmental interactions, such as preventing overgrowth by other microbes [26]. Their presence might be especially important in environments with intricate microbial interactions, such as CCA biofilms. Because of the diverse roles of secondary metabolites, BGCs can be implicated in the synthesis of a wide array of bioactive compounds, with antimicrobials among the most prominent [27]. Consequently, the identification of BGCs in bacterial genomes is an integral part of the bioprospecting process, particularly when the product responsible for antimicrobial traits remains unknown [28].

In this study, CCA-associated bacteria were isolated and tested for antimicrobial properties against antibiotic-resistant and other human and marine pathogens. Selected isolates were subjected to whole-genome sequencing to explore features that might explain their antimicrobial phenotypes. Putative BGCs were then identified, some potentially involved in the production of novel antimicrobial compounds. This study highlights the need to target niche marine environments, such as CCA, for bioprospecting.

## Methods

### Sample collection and taxonomic identification

**Fig. 1.**
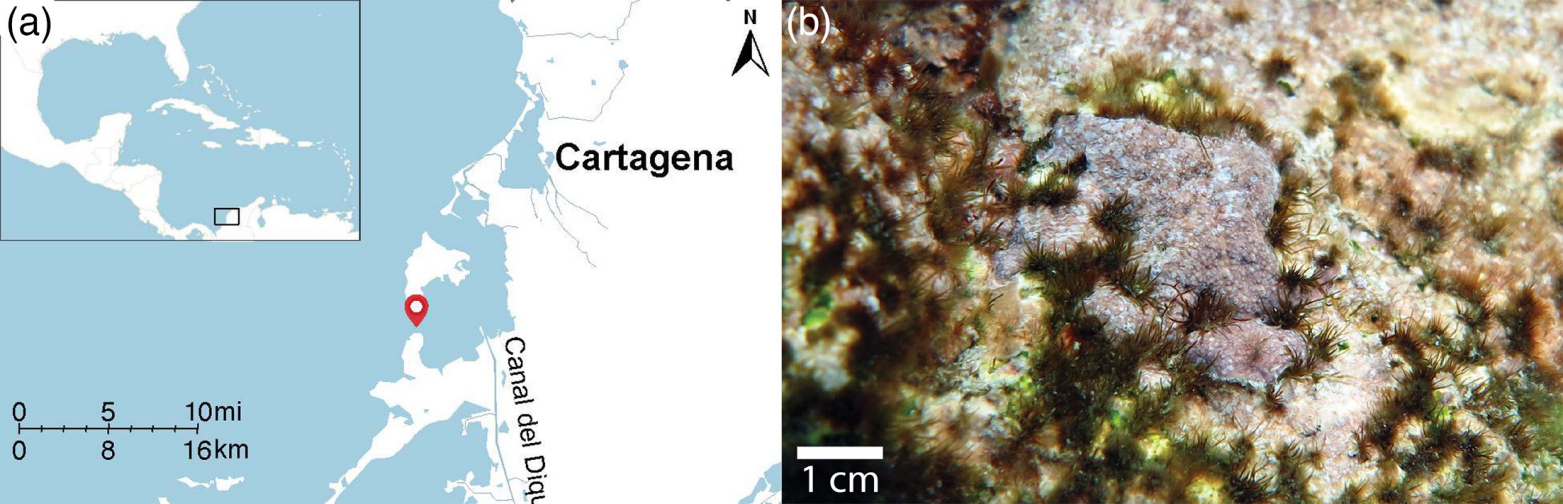
(a) Map displaying the geographic location where the CCA sample was collected. The pin indicates the sampling site, Varadero Reef, Colombia. (b) Underwater photograph of *Hydrolithon* sp. from Islas del Rosario at 3 m depth. Image courtesy of Guillermo Diaz-Pulido (Queensland University of Technology).

The CCA was found in relatively shallow (8 m) coral rubble at the Veradero Reef in the Cartagena Bay, Colombia (10° 18′ 30″ N 75° 34′ 50″ W), on 7 March 2023 (Fig. 1). Samples were collected under the resolutions 0546 (2014) and 094 (2016), issued by the Colombian Ministry of the Environment and re-endorsed in 2019. CCA fragments were stored in sterile 15 ml screw-capped tubes. The CCA exhibited morphological traits characteristic of *Hydrolithon boergesenii*, such as the encrusting and slightly lumpy shape with light purple coloration. Under the light microscope, the thallus appeared dimerous, with a single layer of epithelial cells at the surface and dome-shaped conceptacles, as described in [29]. DNA barcoding using *psbA* as a marker [30,31] was attempted but was unsuccessful due to restricted biological material and the potential presence of PCR inhibitors.

### Bacteria isolation and screening for antimicrobial activity

CCA fragments were swabbed within 3 h of collection using sterile cotton swabs, followed by culture onto marine agar (Difco) plates. Bacteria were subcultured to purification and stored in 96-well plates in 20% glycerol diluted in marine broth. Each of the bacterial isolates was analysed for antibacterial properties against several human and marine pathogens using an agar-overlay assay method (Fig. S1). Culture libraries from 96-well plates were replica plated onto sterile marine agar and grown for 6 days to allow for the production of secondary metabolites. Cultures were then exposed to UV radiation for 1 h to prevent CCA bacteria from cross-contaminating pathogenic test strain overlays. Test strains were grown overnight in 5 ml of strain-specific liquid media (nutrient broth or marine broth, as appropriate) and grown overnight to log phase. Assay plates were overlayed with 10 ml of 0.8% soft agar cooled to 45 °C and inoculated with a standardized amount of test culture as follows: *Bacillus subtilis* (BS; 4 μ ml^−1^, OD595=1.062), *Escherichia coli* (EC; 2 μ ml^−1^, OD595=0.769), *Serratia marcescens* PDL100 (SM; 2.5 μ ml^−1^, OD595=0.801), methicillin-sensitive *S. aureus* (MSSA; 0.5 μ ml^−1^, OD595=0.828), methicillin-resistant *Staphylococcus aureus* (MRSA; 0.5 μ ml^−1^, OD595=0.945), vancomycin-resistant *Enterococcus* (VRE; 4 μ ml^−1^, OD595=1.035) and *Vibrio vulnificus* (VV; 8 μ ml^−1^, OD595=0.808). Zones of growth inhibition were measured in millimetres after 2 days of growth using callipers from the edge of the colony to the edge of the halo; the measurements were done in duplicates (Fig. S1, available in the online Supplementary Material) [9]. Test strains (Table 1) were grown overnight at 37 °C (human pathogens) or 25 °C (marine pathogens) in 5 ml of tryptic soy broth and marine broth, respectively. Aliquots of each broth culture were inoculated into 0.8% agar containing marine or tryptic soy broth, depending on the test strain. CCA library plates were overlaid with ~10 ml of inoculated agar.

**Table 1.** Human and marine pathogen test strains used in antagonistic assays, their antibiotic resistance profiles and associated diseases

Test pathogen	Origin	Resistance	Associated disease
BS	Human	Sensitive	Food spoilage, opportunistic infections
EC	Human	Sensitive	Urinary tract infections, gastroenteritis, septicaemia, meningitis
*S*M	Marine	Sensitive	Urinary tract infections, respiratory infections, septicaemia, wound infections
MSSA	Human	Sensitive	Skin infections, food poisoning, pneumonia, septicaemia, toxic shock syndrome, endocarditis
MRSA	Human	Methicillin	Skin infections, food poisoning, pneumonia, septicaemia, toxic shock syndrome, endocarditis
VRE	Human	Vancomycin	Urinary tract infections, endocarditis, bacteraemia, wound infections, intra-abdominal infections
VV	Marine and human	Sensitive	Severe wound infections, sepsis, foodborne illness, opportunistic marine pathogen

### Genomic DNA extraction and sequencing

Genomic DNA (gDNA) was extracted from 24 h marine agar cultures using the DNeasy Powersoil^®^ Pro Kit (Qiagen, USA) with modifications to the cell lysis steps. Briefly, two to three colonies of each isolate were picked and suspended in 500 µl into the spun PowerBead Pro tubes and mixed with 800 µl of the CD1 solution. Tubes were then placed in a bead-beater vortex and shaken as follows: (1) 5 min at 3,450 r.p.m., (2) a 2 min pause, (3) an incubation for 1 min at 4 °C, (4) 5 min at 3,450 r.p.m. again and (5) a final incubation at 4 °C for 2 min. The bead-beating step replicated settings optimized for DNA extraction from coral-associated bacteria [32], for which the host also precipitates calcium carbonate. After this step, we followed the manufacturer’s instructions. DNA was resuspended in 80 µl of elution buffer. Quantification and quality evaluation were done by electrophoresis in 0.8% agarose gels with 0.5X TBE buffer (45 mM Tris-borate, 45 mM boric acid, 1 mM EDTA, pH 8.2) and using a Qubit fluorometer (Invitrogen^™^). The extracted DNA was further cleaned using a DNA Clean and Concentrator-5 kit (Zymo Research, Irvine, CA, USA) for high-purity absorbance ratios (260/280=1.7 and 260/230=2.2). DNA was then stored at −20 °C until whole-genome sequencing was performed on an Oxford Nanopore MinION Mk1B with no observed degradation prior to the run based on gel electrophoresis. Briefly, high-molecular-weight gDNA libraries were prepared using the Native Barcoding Kit 24 V14 (SQK-NBD114.24, Oxford, UK). Up to 400 ng of gDNA from each isolate was used to achieve equimolar concentration. Then, gDNA end-repair and ligation of native barcodes and adapters were done following the manufacturer’s protocol instructions. The libraries were then pooled and loaded into the primed R10.4.1 flow cell. MinKNOW v23.07.15 was used to monitor sequencing and for demultiplexing. Base calling was conducted separately using Dorado v0.4.3 (github.com/nanoporetech/dorado) implementing the high-accuracy model dna_r10.4.1_e8.2_400bps_sup@v4.2.0 (optimized for Duplex pairs) with default settings other than disabling CUDA acceleration.

### Read processing and genome assembly

Quality of raw reads was assessed using FastQC v0.12.0 (bioinformatics.babraham.ac.uk/projects/fastqc). Adapters were removed, and reads were trimmed using Chopper v0.7.0 [33], removing 100 bp from both 5′ and 3′ ends. Genome size and sequencing depth for each isolate were estimated using Jellyfish v2.3.0 [34] with *k*=25 and GenomeScope 2.0 [35]. *De novo* genome assembly was done using Flye v2.9.2 [36], followed by circularization of complete molecules using Circlator v1.5.5 [37]. Quality of the final assemblies was assessed using CheckM v1.2.2 [38]. To determine whether contigs were of chromosomal or plasmid origin, we aligned each contig against the NCBI nucleotide database using BLASTn v2.16.0 [39]. Taxonomic assignment was done using GTDB-Tk v2.3.2 [40] and the GTDB database release 207 [41] with an average nucleotide identity (ANI) cutoff of ≥95%.

### Gene prediction and functional annotation

Genome annotation was done using Prokka v1.14.5 [42] at default settings. Metabolic pathway analysis was done by first annotating predicted proteins with KEGG Orthology using the blastKOALA web service [43] and then reconstructing metabolic pathways using the online KEGG mapper [44]. COGclassifier v1.0.5 [45] was used to assign predicted proteins into clusters of genes (COGs) with annotated gene functions. COGs were also predicted from the genome of EC strain K-12 substrain MG1655 (NCBI RefSeq accession GCF_904425475) as a reference of a bacterium with minimal known secondary metabolite production [46]. Genes implicated in the production of tetrabromopyrrole (TBP) [47] were manually searched in the Prokka annotations.

### BGC identification and network analysis

BGCs were identified using antiSMASH v7.0.0 [48], DeepBGC v0.1.29 [49] and GECCO v0.9.10 [50]. Circular visualization of genome assemblies and features was done in R using shinyCircos v2.0 [51]. BiG-SCAPE v1.1.9 [52] and the MIBiG database release 2.1 inclusion [53] were used for gene similarity network analyses based on the BGCs identified by antiSMASH, DeepBGC and GECCO. One run was done using all BGCs as input and a sequence similarity cutoff of 70% with the remaining settings at default; networks resulting from this run were considered *conservative*. *Less-conservative* networks were obtained from a second run with the same settings as for the conservative networks, except for the sequence similarity cutoff, which was set to 30%. Clusters resulting from the similarity network analyses were visualized using Cytoscape v3.7.0 [54].

## Results

### CCA-associated bacteria inhibit growth of human pathogens

The 14 bacterial strains isolated from the CCA were found to produce varied zones of inhibition against the test pathogens (Fig. S1), except for the test pathogen *V. vulnificus*, which was consistently inhibited by all 14 isolates (Table 2). Three isolates in particular (CCAH4, CCAH7 and CCAH11) displayed the capacity to inhibit the growth of both Gram-positive and Gram-negative bacteria, with activity against three or more test strains (Table 2). These were selected for subsequent genomic characterization.

**Table 2.** Results of antagonistic assays displaying the inhibitory activity of isolates against various pathogens. Inhibitory zones were measured in millimetres, and duplicate averages are reported

Isolate	**BS**	**EC**	**SM**	MRSA	MSSA	**VRE**	VV
CCAF2	0	0	0	1	0	0	2
CCAF3	0	0	1	0	0	0	2
CCAF4	0	0	1	0	0	0	2
CCAF7	0	0	0	0	0	0	1
CCAF8	0	0	0	0	0	0	2
CCAG11	0	0	0	0	0	0	1
CCAH3	0	0	1	0	0	0	2
CCAH4*	0	0	1	0	1	0	1
CCAH6	0	0	0	0	0	0	2
CCAH7*	0	0	1	0	0	2	2
CCAH8	0	0	0	0	0	0	2
CCAH9	0	0	0	0	0	0	1
CCAH10	0	0	0	0	0	0	1
CCAH11*	2	0	0	7	7	1	2

An asterisk (*) indicates that the corresponding isolate was selected for sequencing.

### Genomic characterization of CCA bacterial isolates

**Fig. 2.**
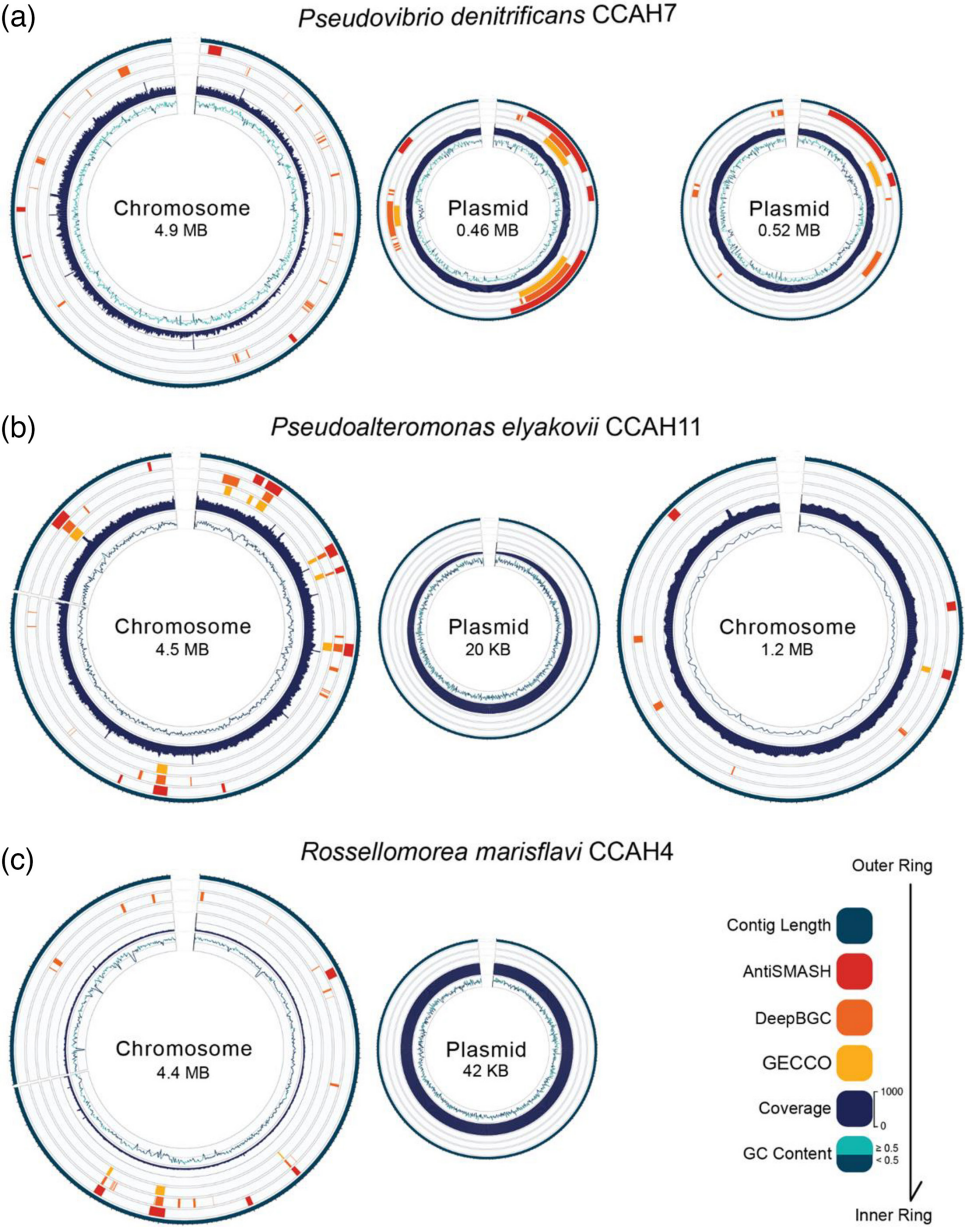
Circular genome representations of *P. denitrificans* CCAH7 (**a**), *P. elyakovii* CCAH11 (**b**) and *R. marisflavi* CCAH4 (**c**), depicting the distribution of BGCs across chromosomes and plasmids. Each representation displays from outer to inner rings: contig length; BGCs identified by antiSMASH, DeepBGC and GECCO; sequencing coverage; and GC content, following the bottom right colour code.

Taxonomic assignment resolved the bacterial isolates to the species level (Fig. S2). Strain CCAH7 was identified as *Pseudovibrio denitrificans* (ANI=95.29%), CCAH11 as *Pseudoalteromonas elyakovii* (ANI=97.29%) and CCAH4 as *Rossellomorea marisflavi* (ANI=98.92%). Genomic features of the three bacterial isolates are summarized in Table 3 and Fig. 2. The genomes were assembled into three to four contigs with N50 lengths ranging from 3.16 to 4.9 Mbp and high completeness (98.64%–100%). All assembled contigs in *P. denitrificans* CCAH7 consisted of entire circular molecules, as did one plasmid contig of *P. elyakovii* CCAH11; no circular molecules were recovered for *R. marisflavi* CCAH4. The predicted protein CDSs ranged between 4,435 and 5,331 per isolate.

**Table 3.** Summary of genome statistics for the sequenced bacterial isolates

Metric	*P. denitrificans*	*P. elyakovii*	*R. marisflavi*
Strain ID	CCAH7	CCAH11	CCAH4
Estimated genome size (bp)	5,573,528	5,498,989	4,887,352
Total assembly length (bp)	5,892,140	5,691,751	4,416,806
Number of contigs	3	4	3
From chromosomes	1	3	2
From plasmids	2	1	1
Number of circularized contigs	3	1	0
Contig N50 length (bp)	4,904,000	3,527,000	3,160,000
G+C content (mol%)	52.42	43.25	46.61
Number of genes	5,331	4,908	4,435
Gene density (%)	86.80	88.10	86.30
Completeness (%)	100.00	100.00	98.68
Number of rRNAs	21	28	27
Number of tRNAs	77	106	100

**Fig. 3.**
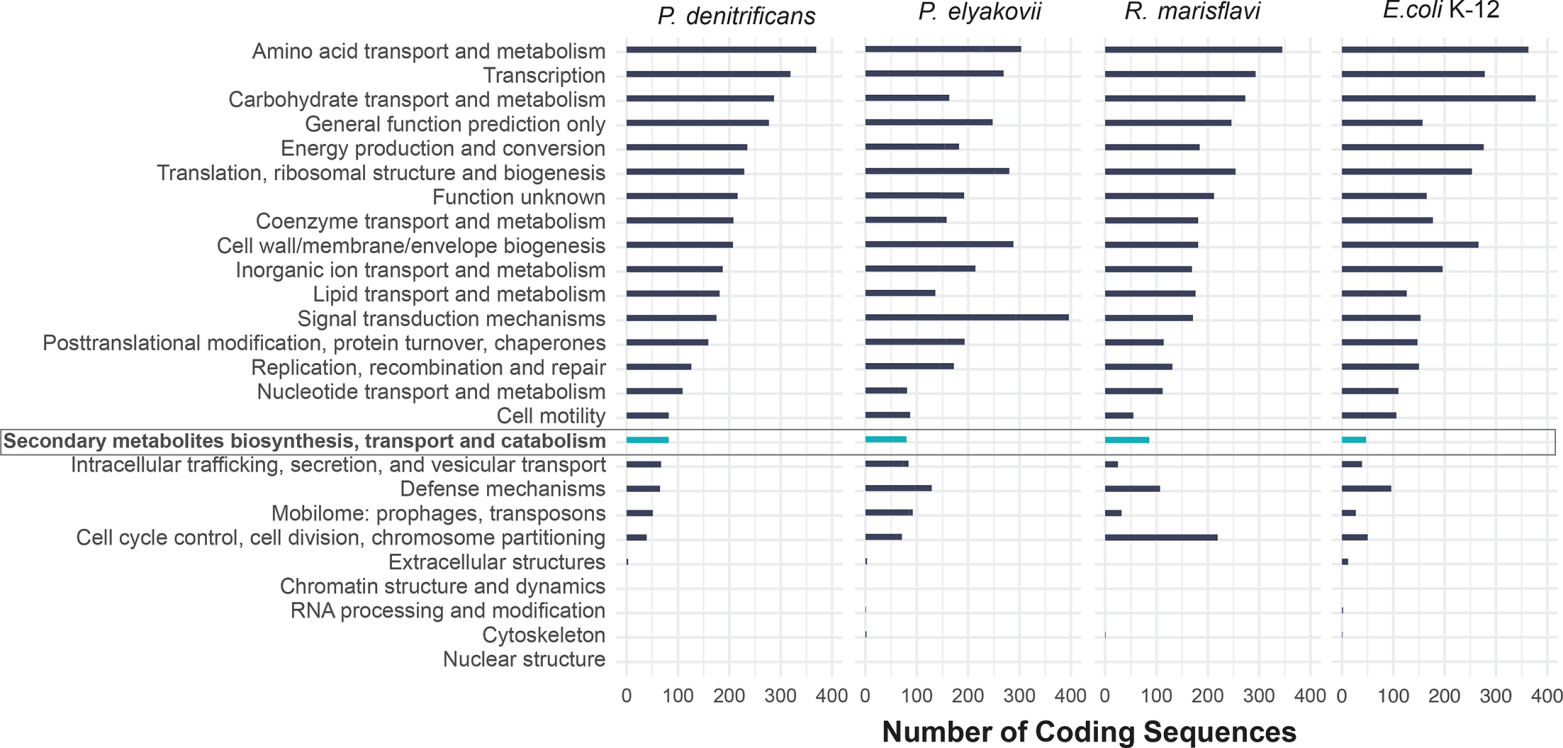
Representation of gene functions in genomes of the selected bacterial isolates and EC strain K-12 substrain MG1655 based on the number of chromosomal protein CDSs in functional categories determined by COGclassifier. The category *Secondary metabolites biosynthesis, transport and catabolism* is highlighted.

COG classification assigned 80.5% of the CDS into functional categories (Fig. 3). The distributions of COG functional categories in chromosomes of *P. denitrificans* CCAH7 and *R. marisflavi* CCAH4 were similar to that of the BGC-deficient *Escherichia coli* K-12 substrain MG1655. *Amino acid transport and metabolism*, *Transcription* and *Carbohydrate transport and metabolism* were the most represented categories in these three genomes, but *Cell cycle control, cell division, chromosome partitioning* was among the least abundant in *P. denitrificans* and *E. coli* and among the most abundant in *R. marisflavi*. The distribution of COG functional categories in *P. elyakovii* was notably different, with *Signal transduction mechanisms*, *Amino acid transport and metabolism* and *Cell wall/membrane/envelope biogenesis* being the most represented. All three isolate genomes harboured substantially more CDS pertaining to *Secondary metabolites biosynthesis, transport and catabolism* in comparison to that of the BGC-deficient *E. coli*. COG functional categories in plasmids of the three isolates were uneven, potentially due to the variable molecule sizes (Fig. S3).

### Potential genomic basis of antimicrobial activity

KEGG metabolic pathway analysis revealed that the genome of *P. elyakovii* contains genes pertaining to *Biosynthesis of siderophore group nonribosomal peptides*; within this pathway, all genes involved in the production of myxochelins A and B were present (Fig. S4). The genome of *R. marisflavi* contained *Carotenoid biosynthesis* genes that suggest the production of lycopene by this bacterium (Fig. S1). The genomes of all three isolates encoded *Streptomycin synthesis* genes, though no isolate contained all genes required for actual streptomycin synthesis (Fig. S6).

**Fig. 4.**
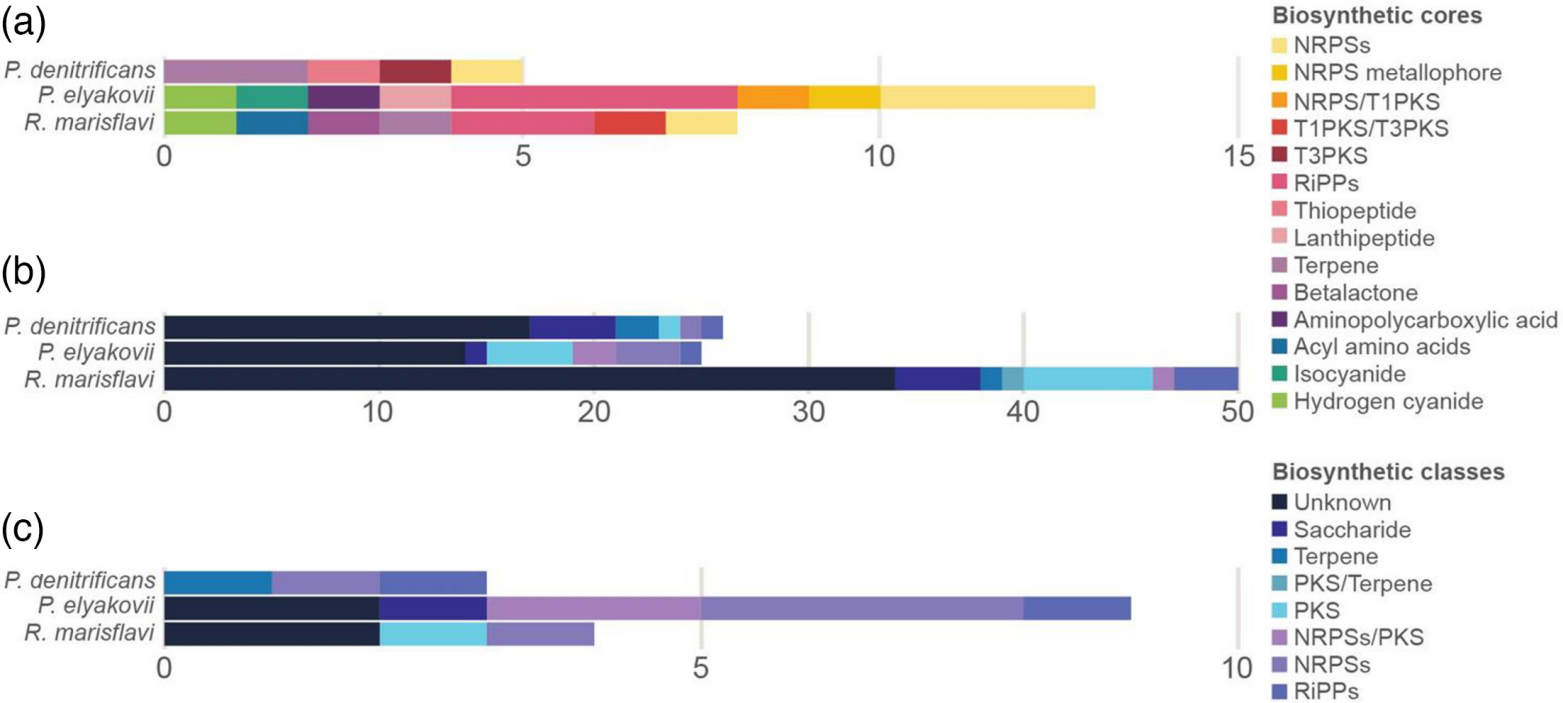
Counts of BGC cores and classes predicted for the three isolates using antiSMASH (**a**), DeepBGC (**b**) and GECCO (**c**).

The three genomes were further investigated to assess their biosynthetic potential in regard to secondary metabolite production. In total, 130 putative BGCs were predicted by several tools (see Methods) with different types of biosynthetic classifications. BGCs identified by antiSMASH were classified into 14 biosynthetic cores (Fig. 4a, Table S1), whereas BGCs identified by GECCO (Table S2) and DeepBGC (Table S3) were classified into five biosynthetic classes, a few pertaining to one or more classes (Fig. 4b, c). Some of the more specific cores found by antiSMASH corresponded to four of the classes found by GECCO and DeepBGC (Fig. 4). Five BGCs had considerable gene similarity (≥50%) to MIBiG BGCs implicated in the production of a range of metabolites (Table 4).

**Table 4.** BGC hits with similarity to MIBiG BGCs and their respective products

Isolate	MIBiG BGC hit	Gene similarity (%)	Product
*P. denitrificans*	BGC0002123	100	Pseudovibriamide A1-6, B1-6
*P. elyakovii*	BGC0002491	100	Myxochelins A and B, pseudochelin A
*P. elyakovii*	BGC0002345	66	Hydrogen cyanide
*P. elyakovii*	BGC0000314	100	Bromoalterochromide A
*R. marisflavi*	BGC0000645	50	Carotenoid

**Fig. 5.**
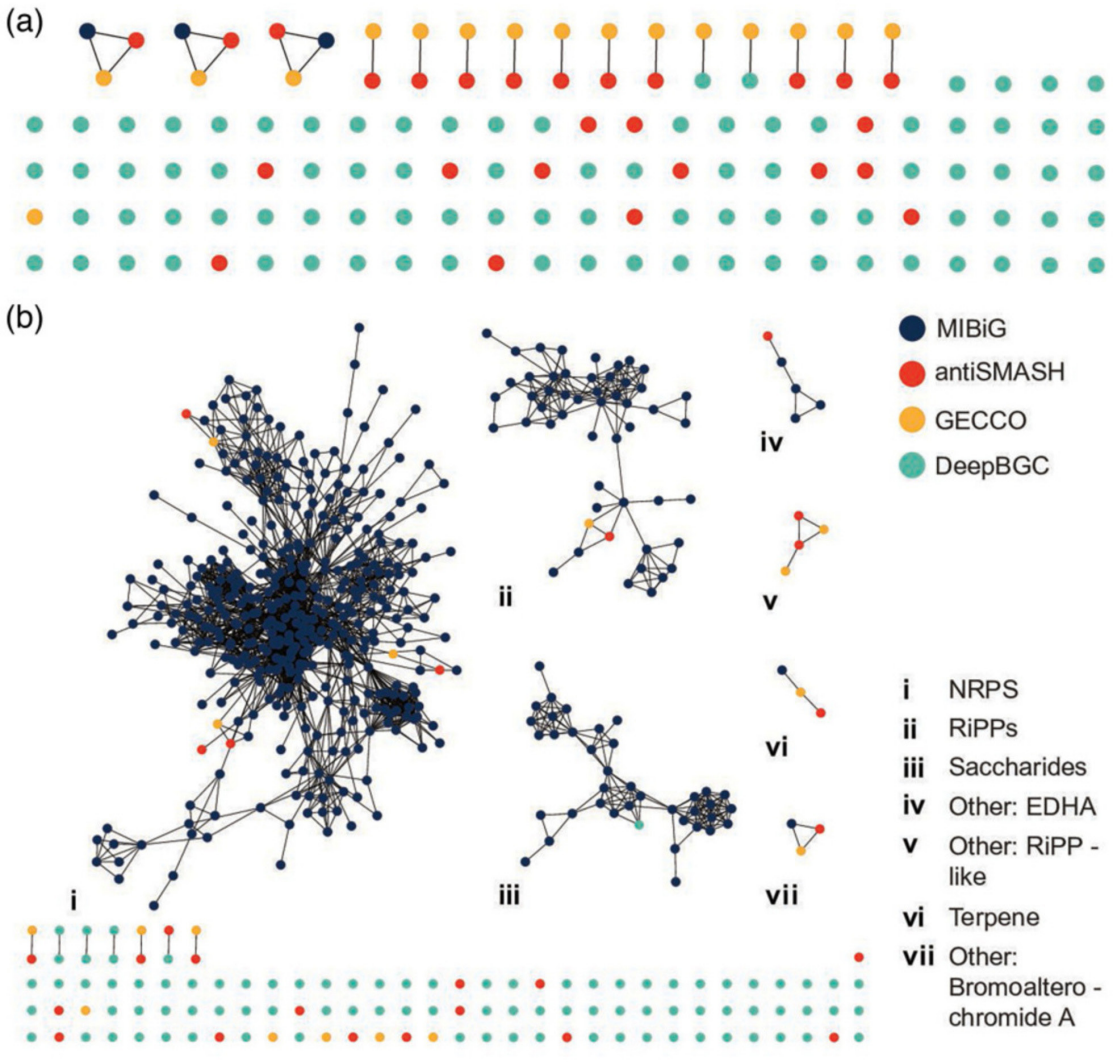
Conservative (a) and less-conservative (b) BGC similarity networks. Nodes represent individual BGCs, coloured by the computational tool used for prediction (see bottom right legend). Edges indicate gene similarity (≥70% for conservative and ≥30% for less conservative) between BGCs. Clusters are labelled (i–vii) according to their predicted product class following the bottom right legend.

The conservative similarity network analysis revealed that most BGCs are unique with no similarity to reference clusters and that there was substantial redundancy between predictions from the different tools (Fig. 5a). Unique BGCs in the similarity network (i.e. singletons) encompassed 100 BGCs. Redundancy between predictions was showcased by 12 pairings that grouped BGCs predicted by different tools but with the same genomic location. Further, three clusters (each containing three BGCs) grouped an MIBiG reference BGC, and an antiSMASH and a GECCO prediction, and the latter two also occurred at the same genomic location. While 14 BGCs grouped with MIBiG clusters in the less-conservative network, most BGCs were still classified as singletons, confirming the little representation of the identified BGCs in the reference database (Fig. 5b). Based on the network analyses and genomic locations of the predicted BGCs, we determined that a total of 115 non-redundant BGCs were predicted from these bacterial genomes: 55 from *P. denitrificans*, 33 from *P. elyakovii* and 27 from *R. marisflavi*; no BGC was shared between isolate genomes.

## Discussion

The present study investigated culturable micro-organisms from a CCA as a first bioprospecting step. Three bacterial isolates exhibited antagonistic activity against several human and marine pathogens, some of which are resistant to commonly used antibiotics. To further assess the antimicrobial potential of these three taxa, their genomes were sequenced. Genes and BGCs potentially underpinning their antimicrobial phenotype were identified. Below, we discuss how these genes and BGCs might be implicated in the antimicrobial activity of these bacteria and in other aspects of their ecology.

### Functional roles of CCA bacterial isolates

The different gene functions and metabolic pathways found in each isolate (Figs 3 and S7) suggest that the isolates occupy distinct niches in the CCA microenvironment. In previous work, *P. denitrificans* has been identified as an important stabilizing agent of reef ecosystems through its coral settlement induction properties [55,56], while simultaneously inhibiting larval settlement of other invertebrates, like barnacles and bryozoans [57]. Coral settlement and metamorphosis have been largely attributed to the secondary metabolite TBP [58]. Nevertheless, no genes (*bmp1-10* [47]) or BGCs involved in the generation of TBP could be found (Table 4). This observation indicates that (i) this particular strain is not capable of inducing coral larvae settlement via this molecule, (ii) the biosynthetic pathways of TBP are not fully known yet or (iii) other inducers yet to be fully characterized might be responsible for this trait [59,60]. Future research on this *P. denitrificans* strain should therefore focus on verifying whether this bacterium can produce TBP and whether it can induce coral settlement, which could lead to the description of new coral larvae settlement inducers.

Various members of the genus *Pseudoalteromonas* have also been shown to induce coral settlement using TBP as a chemical cue [24]. However, like in *P. denitrificans*, no genes involved in TBP production were found in the *P. elyakovii* strain isolated in this study. *P. elyakovii* has been previously found in the mussel *Crenomytilus grayanus* and in the brown seaweed *Laminaria japonica* [61]. This is the first instance, to our knowledge, that this bacterium has been isolated from a member of the phylum Rhodophyta. Within *Laminaria*, *P. elyakovii* has been isolated from spot-wounded fronds, suggesting that *P. elyakovii* may be causing damage to this alga. Further, our analyses of metabolic pathways (Fig. S4) and BGCs (Table 4) suggest that this isolate may be actively acquiring iron from the environment and/or host through siderophores like myxochelins A and B, enterochelin and pseudochelin A. Iron acquisition mechanisms drive potential virulence of well-established pathogens [62,63]. This bacterium might thus be an algal pathogen.

The genome of *R. marisflavi* contained genes implicated in the resistance to arsenic (i.e. *arsB*, *arsC* and *arsR*) and in the production of the antioxidant carotenoid lycopene (Table 4 and Fig. S1), which is consistent with the pink colour of the colonies grown in agar plates. A genomic characterization of another member of the same genus, *Rossellomorea* sp. y25, found similar metabolic capabilities, with added resistance to cadmium and zinc [64]. These genetic features were hypothesized to play a role in the adaptation to deep-sea environments, where this bacterium was isolated from. In *R. marisflavi*, resistance to heavy metals might be associated with the potential for bioaccumulation of trace elements in CCA [65]. Though we are not aware of any reports of arsenic in these algae, contamination with heavy metals has occurred several times in Cartagena Bay [66,69], where the CCA analysed here was sampled. Lycopene production, on the other hand, might be related to its ability to prevent the lethal action of UV radiation on bacterial cells [70] given the high-light environments where CCA inhabit.

### Antimicrobial activity is likely due to BGCs with unknown functions

Metabolic pathway exploration showed no prominent representation of genes involved in described KEGG antibiotic biosynthesis pathways in either of the three CCA isolates (Fig. S8). However, the genomes of all three isolates contained several of the genes implicated in the biosynthesis of streptomycin, which could result in the community-based production of this antibiotic (Fig. S6). Though metabolic complementation [71] among members of the CCA microbiome might be a mechanism leading to the biosynthesis of some antimicrobials, it is not possible that the observed antimicrobial activity is a result of community-based biosynthesis, as we tested the isolates separately.

Antimicrobial activity is often a result of products of BGCs. The most frequent BGC classes identified in this study included non-ribosomal peptides, ribosomally synthesized and post-translationally modified peptides, polyketides, saccharides and terpenes (Fig. 1). These biosynthetic classes are commonly identified in bioprospecting studies of prokaryotes in marine environments [72]. Nevertheless, the number of BGCs recovered in the genomes of the three isolates studied here was unusually high. For example, BGCs predicted by antiSMASH ranged between 5 (in *R. flavimaris*) and 13 (in *P. elyakovii*), whereas a former large-scale study predicted 18,043 BGCs from 5,743 microbial genomes (i.e. on average 3.14 BGCs per genome) [73]. In addition, the number of CDS involved in the production of secondary metabolites across all three genomes was nearly twice as high as that in the BGC-deficient *E. coli*, indicating a high potential for biosynthesis of such metabolites (Fig. 3).

Only five BGCs could be annotated with a known function (Table 4), from which three were predicted to produce known secondary metabolites. One of the BGCs, identified in the genome of *P. denitrificans*, is involved in the synthesis of pseudovibriamides A1-6 and B1-6, which are hypothesized to play a role in surface motility [74]. The other two BGCs with known function were found in the genome of *P. elyakovii*. One of them is implicated in the production of catecholate-type siderophores, like myxochelins A and B, and pseudochelin A. While these siderophores are crucial for bacterial iron homeostasis [75], they also exhibit weak activity against Gram-positive bacteria [76]; yet *P. elyakovii* inhibited both Gram-positive and -negative pathogens in antagonistic assays. The other BGC is associated with the production of bromoalterochromide A. *Pseudoalteromonas* bromoalterochromides often have antibacterial activity [77], but no antimicrobial properties have been observed in bromoalterochromide A [78]. The carotenoid lycopene found in *R. marisflavi* (Table 4 and Fig. S5) has been shown to have antimicrobial activity against Gram-negative bacteria, specifically against *E. coli* [79], and members of the Chlamydiaceae family, such as *Chlamydia trachomatis* and *Chlamydia pneumoniae* [80]. While this carotenoid could explain some of the antimicrobial activity observed in the antagonistic assays, the *R. marisflavi* strain here did not inhibit EC, suggesting that *R. marisflavi* was not producing lycopene during the assays despite having the genomic capability. Therefore, the observed antimicrobial phenotypes are unlikely due to these BGCs.

Though biosynthetic pathways and known BGCs might underpin some of the antimicrobial activity revealed by the antagonistic assays, we hypothesize that this activity is most likely attributed to BGCs with unknown function. Given that most characterized biosynthetic pathways and BGCs have already been extensively explored for commercial antimicrobial production, these known compounds likely contribute to the existing resistance profiles of the tested pathogens. Hence, we believe that unknown metabolites are the ones that the tested pathogens have not been commonly exposed to and are therefore susceptible to. BGCs can produce a wide range of metabolites [26], many of which remain to be explored; therefore, they could be responsible for this observed antimicrobial activity. Of special interest are the singletons, unique BGCs that appear only once in our dataset and show no significant similarity to known clusters. These singletons are particularly promising candidates for novel antimicrobial discovery as they represent previously uncharacterized biosynthetic targets, though we cannot disregard the possibility that some of these BGCs could be prediction artefacts [81].

### Future directions

The advancement of bioprospecting efforts largely relies on deepening our understanding of fundamental microbial ecology. Certain microhabitats may be more prone to harbouring novel antimicrobials, yet it remains difficult to identify which to target for bioprospecting. This knowledge gap extends beyond the ecology of micro-organisms to the genomic basis of natural products, such as BGCs. Many BGCs remain uncharacterized, and their prediction from genomic data is based on similarity to other known BGCs, which hinders bioprospecting efforts, as this study highlights.

One priority moving forward should be the consistent curation and functional characterization of unknown BGCs. To further the breadth of BGCs we identify, effort should be placed in employing multiple complementary approaches rather than relying on a single tool. This strategy allows for more comprehensive identification of BGCs that might be missed when using a single method. New methods that expedite the bioprospecting pipeline are also warranted. In this study, we employed several methods to identify BGCs of interest and demonstrated how network analyses can effectively prioritize promising BGCs by comparing their similarity to previously characterized clusters. This approach enables more targeted investigation of potentially novel compounds while reducing the resources spent on redundant or less promising BGCs. Though costly, the development and enrichment of BGC databases and their continuous curation will provide an essential foundation for more targeted and efficient antimicrobial (and other natural products) discovery efforts.

In summary, our study emphasizes the potential of CCA as valuable targets for antimicrobial discovery. We highlight the wide range of metabolic potential of CCA-associated bacteria through metabolic pathway analysis and the identification of 115 non-redundant BGCs. From these, 97 singleton BGCs represent promising targets for unlocking novel biosynthetic potential in CCA-associated bacteria.

## Supplementary material

10.1099/mgen.0.001456 Uncited Supplementary Material 1.

10.1099/mgen.0.001456 Uncited Supplementary Material 2.

## Abbreviations

ANI: average nucleotide identity
BGC: biosynthetic gene cluster
BS: *Bacillus subtilis*
CCA: crustose coralline algae
COGs: clusters of genes
EC: *Escherichia coli*
gDNA: genomic DNA
MRSA: methicillin-resistant *Staphylococcus aureus*
MSSA: methicillin-sensitive *Staphylococcus aureus*
SM: *Serratia marcescens*
TBP: tetrabromopyrrole
VRE: vancomycin-resistant *Enterococcus*
VV: *Vibrio vulnificus*
