# Supplementary material for: Genomic potential of crustose coralline algae-associated bacteria for the biosynthesis of novel antimicrobials
Source: Microb Genom. 2025 Jul 25;11(7):001456. doi: 10.1099/mgen.0.001456 (PMC12452183; doi:10.1099/mgen.0.001456)
Supplement: Uncited Supplementary Material 1. [file mgen-11-01456-s001.pdf]

Genomic Potential of Crustose Coraline Algae-Associated Bacteria for the Biosynthesis of Novel Antimicrobials

Diego Lera-Lozano[1,2], Jordan S. Ruiz-Toquica[3,4], Samantha A. Kratman[1,5], Matthew W. Holt[6], Clancy A. McIntyre[1], Elizabeth K. Jones[6], Mateo López-Victoria[7], Kim B. Ritchie[6], Mónica Medina[1,2], Raúl A. González-Pech[1,8]

[1]Department of Biology, The Pennsylvania State University, University Park, Pennsylvania, USA

[2]Department of Ecology and Evolutionary Biology, University of California Los Angeles, Los Angeles, California, USA

[3]Faculty of Natural Sciences and Engineering, Universidad de Bogotá Jorge Tadeo Lozano, Bogota, Colombia

[4]Department of Biology and Marine Biology, University of North Carolina Wilmington, Wilmington, North Carolina, USA

[5]Schreyer Honors College, The Pennsylvania State University, University Park, Pennsylvania, USA

[6]Department of Natural Sciences, University of South Carolina Beaufort, Beaufort, South Carolina, USA

[7]Department of Natural Sciences and Mathematics, Pontificia Universidad Javeriana, Cali, Colombia

[8]Department of Biology, Texas State University, San Marcos, Texas, USA

[*]Address correspondence to dfl5440@psu.edu or r.gonzalezpech@txstate.edu.

SUPPLEMENTARY FIGURES

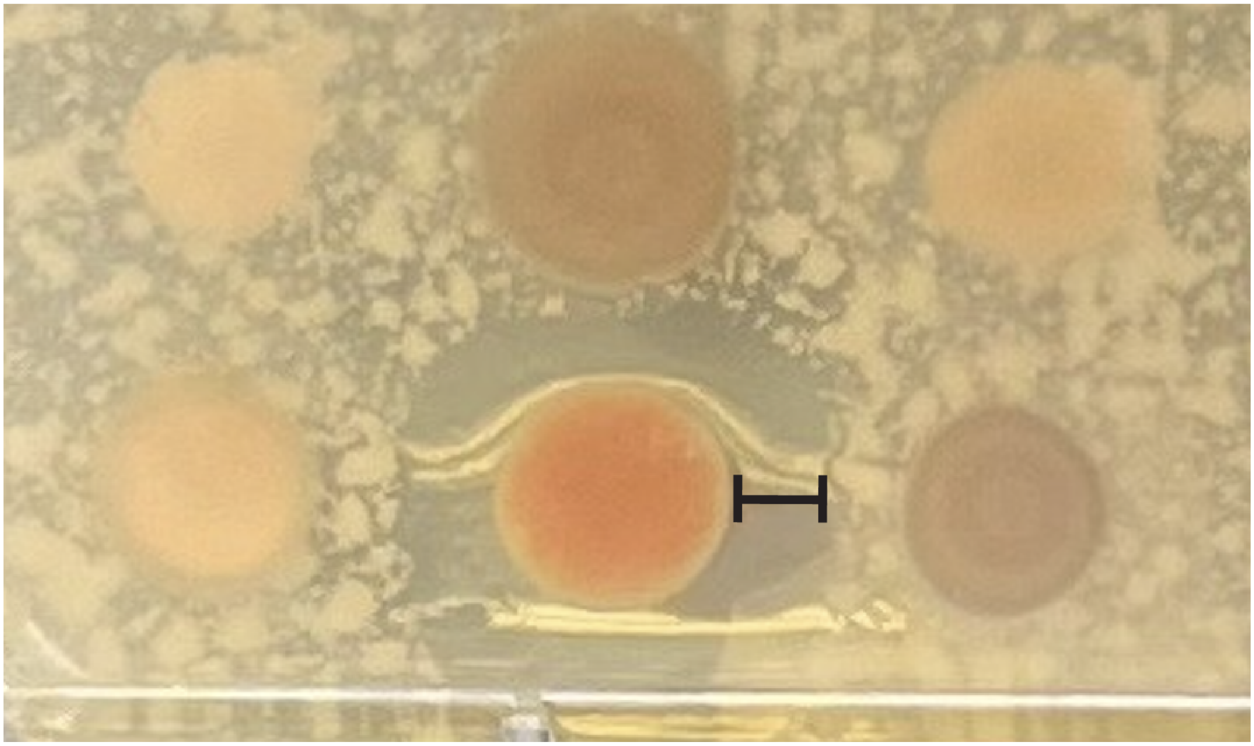

SUPP FIG 1. Example of inhibition halo produced by the agar-overlay assay of *Pseudoalteromonas elyakovii* CCAH11 against methicillin-resistant *Staphylococcus aureus* (MRSA). The bar denotes the value measured and reported in Table 2.

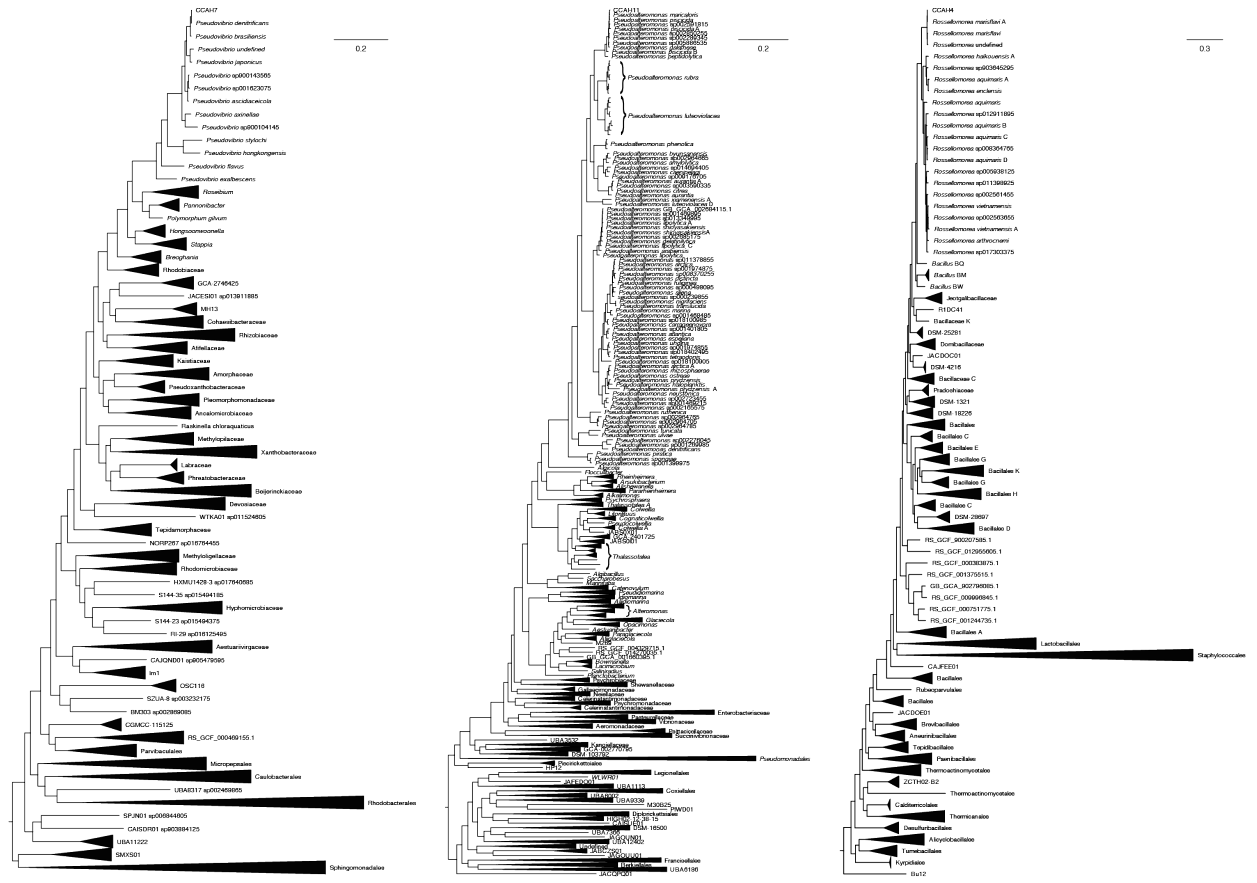

SUPP FIG 2. Phylogenetic placement of *Pseudovibrio denitrificans* CCAH7 (left), *Pseudoalteromonas elyakovii* CCAH11 (middle), and *Rossellomorea marisflavi* CCAH4 (right). Trees were produced from the genomic assessment run by GTDB-Tk (see Methods).

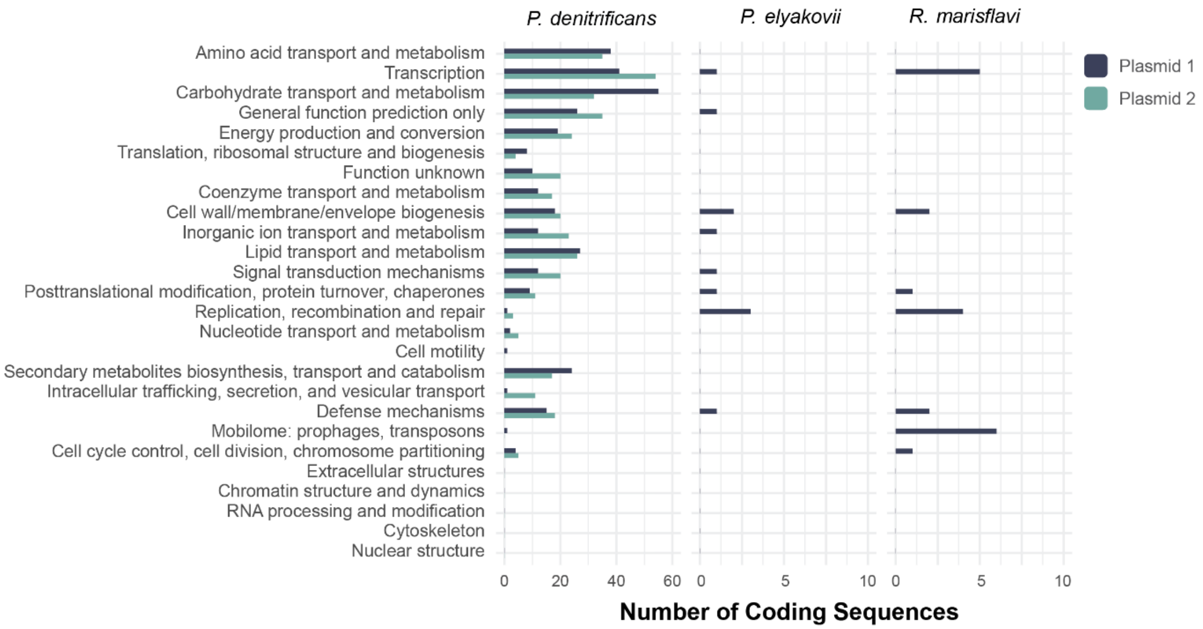

SUPP FIG 3. Number of plasmid protein-coding sequences (CDS) in functional categories determined by COGclassifier. Note the different scales across panels.

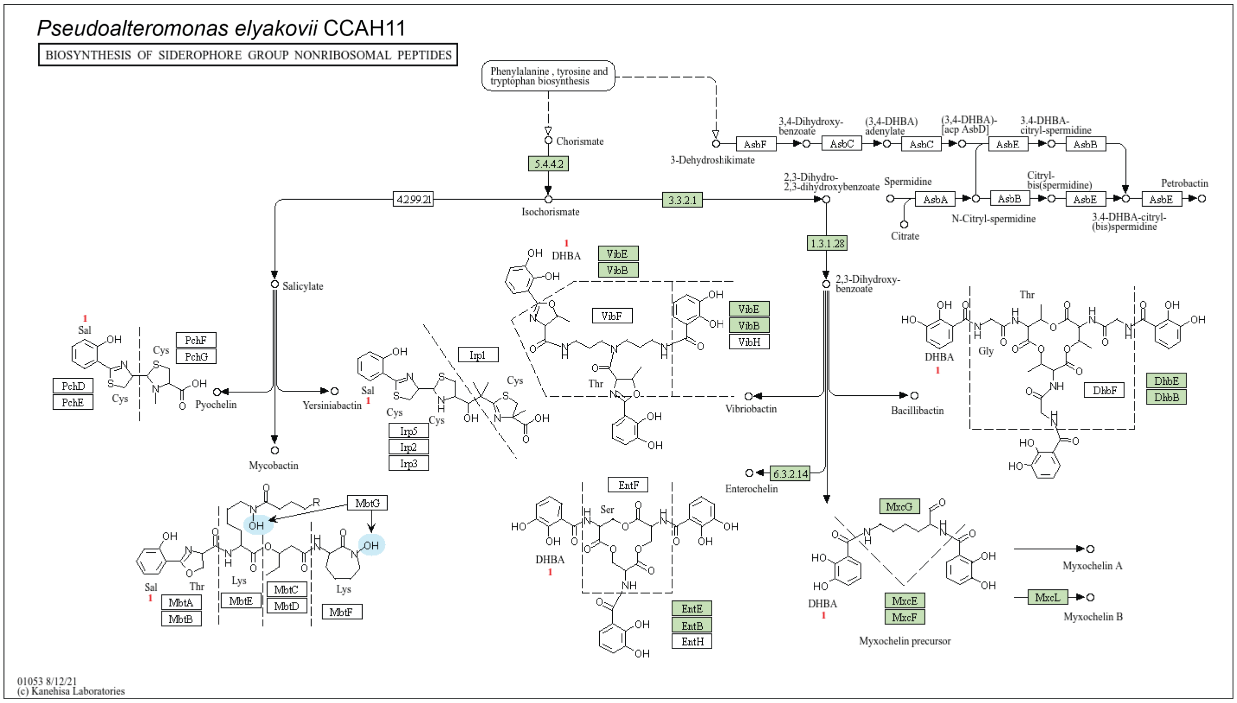

SUPP FIG 4. KEGG pathway diagram of the biosynthesis of siderophore group non-ribosomal peptides. Green boxes represent genes present in the genome of *Pseudoalteromonas elyakovii* CCAH11.

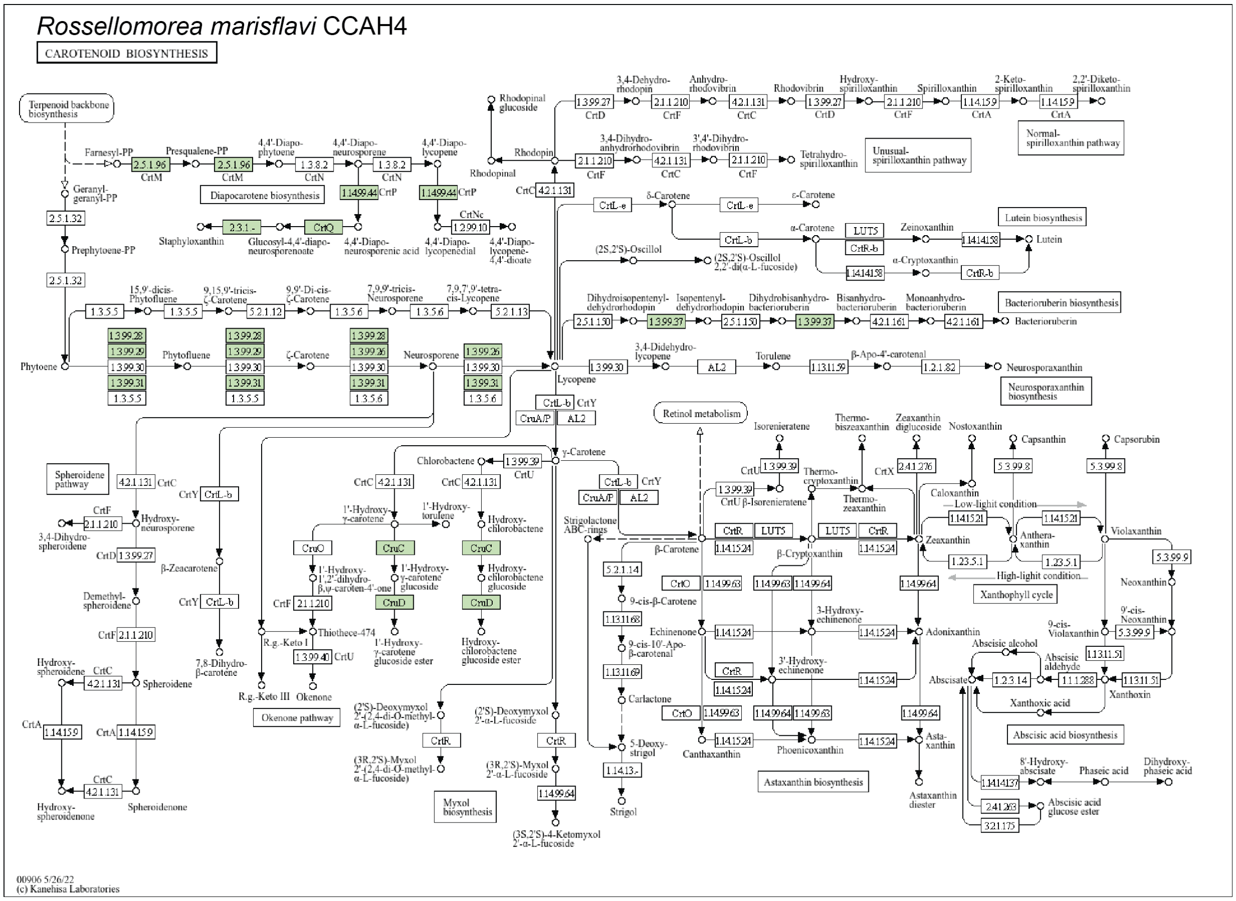

SUPP FIG 5. KEGG pathway diagram of the biosynthesis of carotenoids. Green boxes represent genes present in the genome of *Rossellomorea marisflavi* CCAH4.

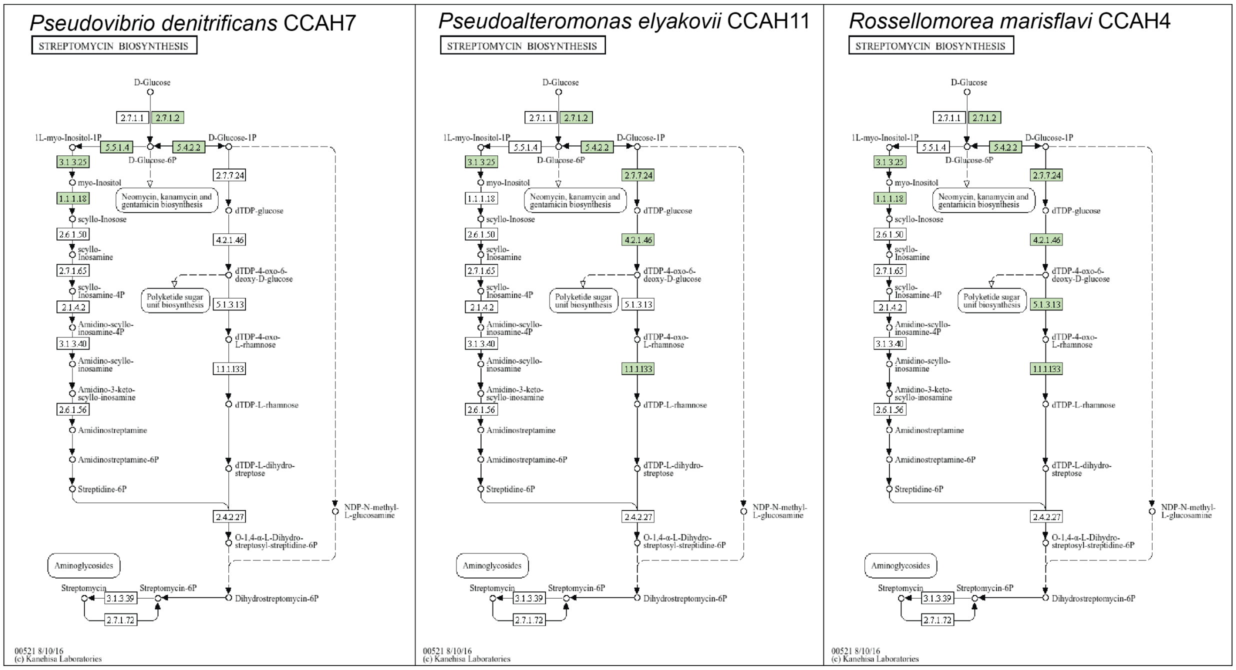

SUPP FIG 6. KEGG pathway diagram of the biosynthesis of streptomycin. Green boxes represent genes present in the genomes of *Pseudovibrio denitrificans* CCAH7 (left), *Pseudoalteromonas elyakovii* CCAH11 (middle), and *Rossellomorea marisflavi* CCAH4 (right).

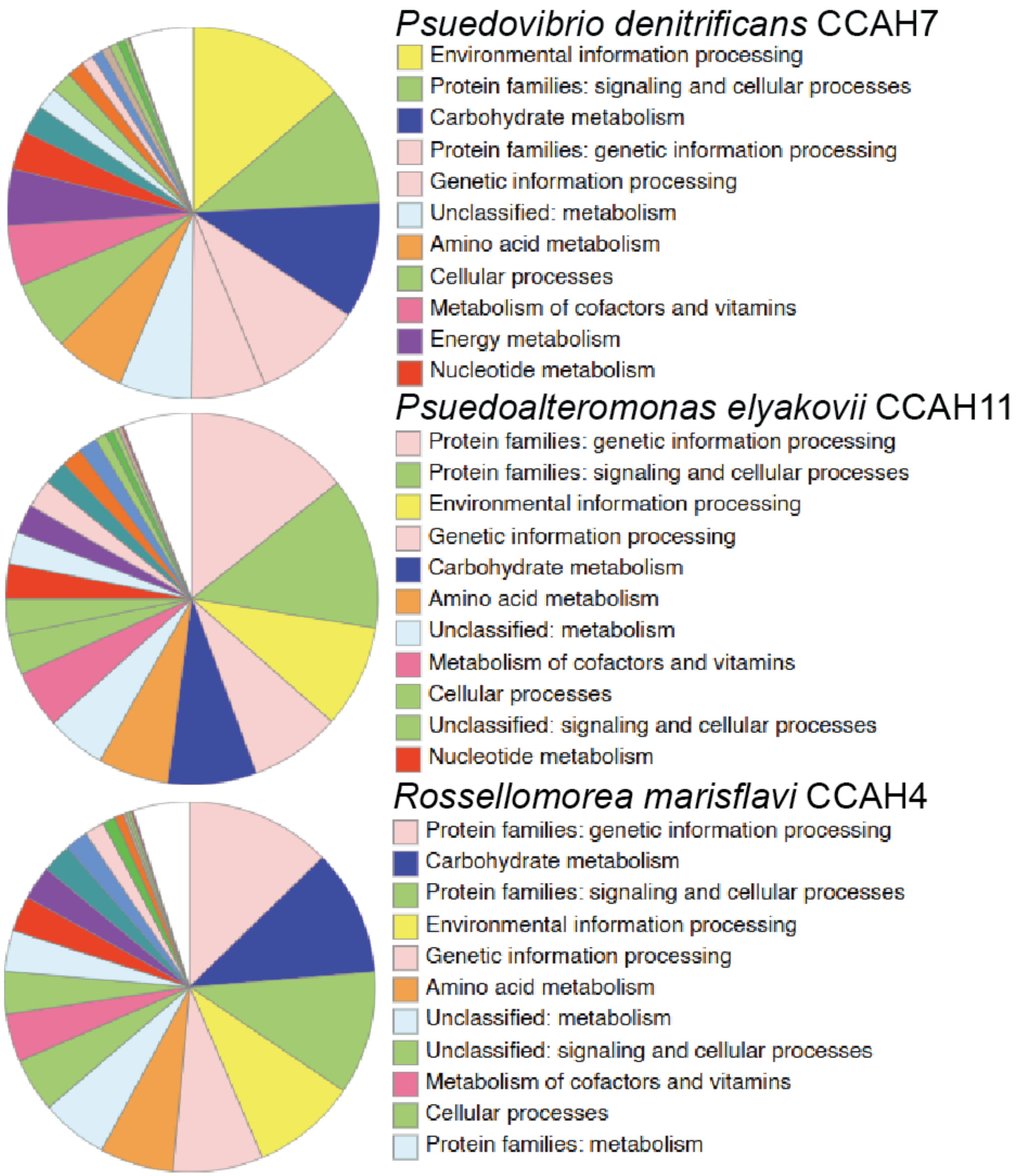

SUPP FIG 7. Metabolic pathway representation in the genomes of *Pseudovibrio denitrificans* CCAH7 (top), *Pseudoalteromonas elyakovii* CCAH11 (middle), and *Rossellomorea marisflavi* CCAH4 (bottom), as determined by blastKOALA annotation.

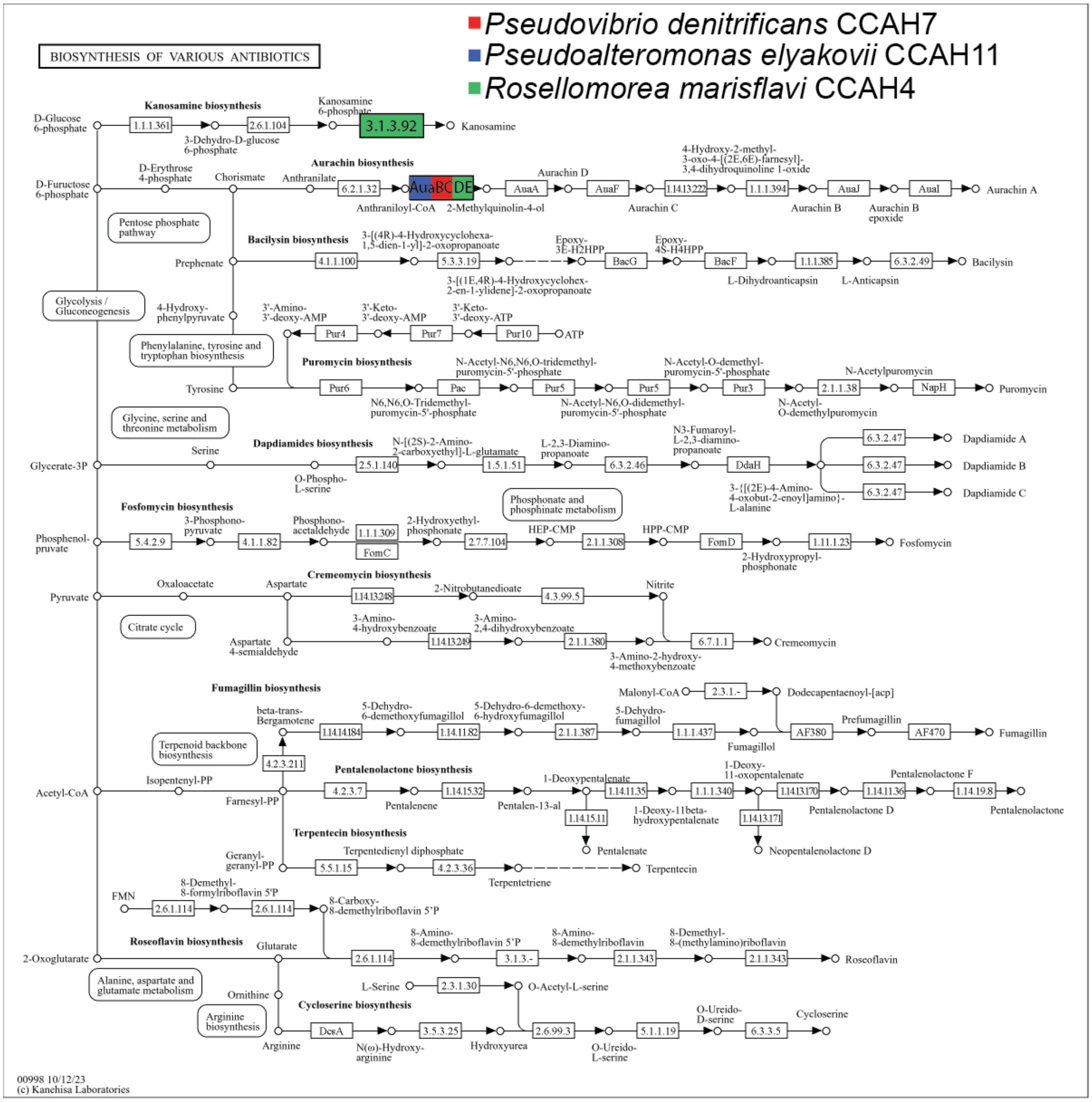

SUPP FIG 8. KEGG pathway diagram of the biosynthesis of various antibiotics. Red boxes represent genes present in the genome of *Pseudovibrio denitrificans* CCAH7. Blue boxes represent genes present in the genome of *Pseudoalteromonas elyakovii* CCAH11. Green boxes genes represent in the genome of *Rossellomorea marisflavi* CCAH4.
